# LGBTQ-RELATED DISCRIMINATION REDUCES SLEEP QUALITY IN OLDER ADULTS: EVIDENCE FROM VUSNAPS AND UCNETS

**DOI:** 10.1093/geroni/igad104.0184

**Published:** 2023-12-21

**Authors:** Tara McKay, Nathaniel Tran, Lana Trautman, Kirsty Clark

**Affiliations:** Vanderbilt University, Nashville, Tennessee, United States; Vanderbilt University, Nashville, Tennessee, United States; Vanderbilt University, Nashville, Tennessee, United States; Vanderbilt University, Nashville, Tennessee, United States

## Abstract

Sleep is a critical protective factor for health outcomes with low-quality sleep linked to increased risk of diabetes, cardiovascular disease, and cognitive decline. Theoretical work proposes that minority stress may negatively affect sleep quality for LGBTQ and other minoritized populations. We examine differences in sleep quality across sexual orientation and gender identity and test the relationship between minority stress and sleep quality for LGBTQ adults. Data come from 1) UC Berkeley Social Networks Study (UCNets), a panel study of 50–70-year-olds with an oversample of older LGBTQ adults and 2) the Vanderbilt University Social Networks, Aging, and Policy Study (VUSNAPS), a panel study of older LGBTQ adults aged 50-76. LGBTQ identity includes participants who are transgender and/or gay, lesbian, bisexual, or something else. We estimate Poisson models predicting sleep quality as a function of LGBTQ identity and exposure to LGBTQ-related discrimination. Sleep quality is measured using three items available in both studies: 1) the number of nights per week trouble falling asleep, 2) number of nights per week trouble staying asleep, and 3) number of mornings per week waking well rested. Experiences of LGBTQ-related discrimination in the last year were measured using the Daily Heterosexist Experiences Questionnaire. We find that LGBTQ identity is significantly associated with poorer sleep quality compared to non-LGBTQ peers. Among LGBTQ adults, sleep quality declines as the number and intensity of recent experiences of LGBTQ-related discrimination increases. Poorer sleep quality may be an underlying mechanism exacerbating health disparities across sexual orientation and gender identity.

